# Mindfulness training for stress management: a randomised controlled study of medical and psychology students

**DOI:** 10.1186/1472-6920-13-107

**Published:** 2013-08-13

**Authors:** Michael de Vibe, Ida Solhaug, Reidar Tyssen, Oddgeir Friborg, Jan H Rosenvinge, Tore Sørlie, Arild Bjørndal

**Affiliations:** 1 Norwegian Knowledge Centre for the Health Services, P.O. Box 90153, N-0130 Oslo, Norway; 2 Department of Psychology, Faculty of Health Sciences, University of Tromsø, N-9037 Tromsø, Norway; 3 Department of Behavioural Sciences in Medicine, Institute of Basic Medical Sciences, Faculty of Medicine, University of Oslo, P.O. Box 1111, N-0317 Oslo, Norway; 4 Psychiatric Research Center of Northern Norway, University Hospital of Northern Norway, N-9291 Tromsø, Norway; 5 Department of Clinical Medicine, Faculty of Health Sciences, University of Tromsø, N-9037 Tromsø, Norway; 6 Department of General Psychiatry, University Hospital of Northern Norway, N-9291 Tromsø, Norway; 7 Center for Child and Adolescent Mental Health, Eastern and Southern Norway, P.O. Box 4623, N-0405 Oslo, Norway

**Keywords:** Stress management, Mental distress, Well-being, Five facet mindfulness questionnaire, Gender differences, Undergraduate medical and psychology education

## Abstract

**Background:**

Distress and burnout among medical and psychology professionals are commonly reported and have implications for the quality of patient care delivered. Already in the course of university studies, medicine and psychology students report mental distress and low life satisfaction. There is a need for interventions that promote better coping skills in students in order to prevent distress and future burnout. This study examines the effect of a seven-week Mindfulness-Based Stress Reduction (MBSR) programme on mental distress, study stress, burnout, subjective well-being, and mindfulness of medical and psychology students.

**Methods:**

A total of 288 students (mean age = 23 years, 76% female) from the University of Oslo and the University of Tromsø were randomly allocated to an intervention or control group. The control group continued with their standard university courses and received no intervention. Participants were evaluated using self-reported measures both before and after the intervention. These were: the ‘General Health Questionnaire, Maslach Burnout Inventory Student version, Perceived Medical School Stress, Subjective Well-being, and Five Facet Mindfulness Questionnaire’ and additional indices of compliance.

**Results:**

Following the intervention, a moderate effect on mental distress (Hedges’g 0.65, CI = .41, .88), and a small effect on both subjective well-being (Hedges’g 0.40, CI = .27, .63) and the mindfulness facet ‘non-reacting’ (Hedges’g 0.33, CI = .10, .56) were found in the intervention group compared with the control group. A higher level of programme attendance and reported mindfulness exercises predicted these changes. Significant effects were only found for female students who additionally reported reduced study stress and an increase in the mindfulness facet ‘non-judging’. Gender specific effects of participation in the MBSR programme have not previously been reported, and gender differences in the present study are discussed.

**Conclusion:**

Female medical and psychology students experienced significant positive improvements in mental distress, study stress, subjective well-being and mindfulness after participating in the MBSR programme.

**Trial registration:**

NCT00892138

## Background

Distress among medical and psychology professionals is associated with poorer patient care [1], a higher risk of future medical errors [2], as well as depression, anxiety and reduced life satisfaction [3-5]. Whether such problems can be prevented through stress-reducing interventions for psychology and medical students has not yet been fully investigated, and there are noticeably few studies involving psychology students within this area of research. In Norway, admission criteria to both the medicine and psychology professional study are very high. Medical and psychology students are typically resourceful high achievers who are able to cope with the challenges of professional study yet they also commonly report mental distress and low levels of life satisfaction [6-8]. A review of the distress experienced by medical students has emphasised the need for studies that contribute to a better understanding of how to promote well-being [9]. A failure to promote well-being may lower academic performance [10,11]. Other studies have addressed the need to prevent future potential stress and burnout through the teaching of better coping skills to students [8,12,13]. There is currently a shortage of well-designed and effective intervention studies to address such challenges.

Mindfulness-Based Stress Reduction (MBSR) has been used increasingly over the last 30 years to help people cope with physical and mental distress. By cultivating an open, accepting attitude within the present moment towards internal and external experiences, MBSR training has been shown to reduce mental distress and promote well-being in both clinical and non-clinical populations [14]. Previous studies of mindfulness training given to medical students in the United States of America (USA) and Australia have reported beneficial outcomes [15-18]. Few studies of mindfulness training have been undertaken on psychology students [19,20]. Although these studies have indicated similar beneficial results they have suffered from both poor statistical strength and inadequate randomisation and this has limited the validity of their conclusions. To date, there has also been a lack of studies comparing the effects of mindfulness-based interventions on medical and psychology students as well as multi-site studies which could facilitate the generalisation of results.

Many studies have indicated that women report higher levels of distress and lower levels of subjective well-being than men [21-23] but the field is still characterised by a lack of attention to gender-specific effects. A meta-analysis of 31 randomised controlled MBSR trials identified only two studies that had analysed gender as a moderator variable and neither of these reported gender-specific effects [14].

There is a growing body of research indicating that MBSR programmes lead to increased levels of participant self-reported mindfulness [14] but such findings have not yet been confirmed in a randomised controlled study of students. In studies of the effects of MBSR programmes, moderator variables such as course attendance and mindfulness practice have also been examined in several studies but the results have been mixed [14]. This may be due to variations in the power of such studies to detect effects [24].

Our study aimed to evaluate the effects of a seven-week MBSR programme in a student sample from two Norwegian universities. The study had three main aims: first, to test the hypothesis that the MBSR programme would enhance mental health among medical and psychology students as measured by multiple dimensions of psychological health and well-being. Second, we aimed to test whether the intervention effects were influenced by gender, the university courses (psychology or medicine), the university locations, course instructors, intervention participation and self-reported mindfulness practice. Finally, we aimed to assess our expectation that the MBSR intervention would increase facets of mindfulness.

## Methods

### Participants and recruitment

In 2009 and 2010, medical and psychology students in their second or third term at the University of Oslo and the University of Tromsø respectively, were invited to participate in the study. Information was provided during classes by the study project managers followed by an email inviting people to visit a website for more information and the opportunity to sign up for the study. Informed consent was obtained electronically after which the participants completed an online questionnaire (T1). Because the programme purpose was health promotion and stress management rather than psychotherapy, no exclusion criteria were used and the students were not screened for mental illness. The sample size was calculated based on an expected reduction in mental distress and perceived medical school stress of 20% in the intervention group, and on longitudinal studies of how stress and mental health problems increase during university programmes among Norwegian medical students [25,26]. 60–100 participants per study group were needed for the power calculation (alpha level .05, 80% power) to test whether the intervention could prevent such increases. The study protocol is available at www.clinicaltrials.gov [27], where further details on sample calculation can be found.

### Procedures

After the participants completed the T1 questionnaire, a computer program (a Java-based random number generator) was used to randomly assign students either to the intervention group or to the control group. The randomisation was performed separately for each class of students without stratification by gender. An email message sent two weeks prior to the intervention informed the study participants of their group allocation. Within the two weeks after the intervention, participants were asked to complete a second questionnaire (T2) and they received up to three email reminders to prompt them to do so. The head technician at the Norwegian Knowledge Centre for the Health Services assigned each participant an identity (ID) number which was then assigned to their online questionnaires to ensure that the data remained anonymous. Only the head technician had access to data that showed the link between the student identities and the ID numbers, and he was not involved in the study in any other way.

To compensate study participants for using approximately 40 minutes to complete the T1 and T2 questionnaires each time and to reduce potential drop-out rates, those students who took part in the study received a book voucher after they had completed the T2 questionnaire. The study was approved by the Regional Committee for Medical and Health Research Ethics in Norway, and by the Norwegian Data Inspectorate.

#### Description of the intervention

The MBSR programme – based on the programme developed by Kabat-Zinn [28] – was conducted independently of the students’ study curricula and lasted seven weeks. The original programme consisted of eight weekly sessions of 2.5 hours each, a 7-hour session that took place between week six and seven and 45 minutes of formal mindfulness practice at home. However, information obtained from the focus group interviews with students prior to the study led to the programme being reduced to six weekly sessions of 1.5 hours each, a 6-hour session in week seven, and 30 minutes of daily home mindfulness practice. Apart from these changes, the intervention was equivalent to the original MBSR programme.

The MBSR programme used in this study consisted of: 1) physical and mental exercises to increase participant mindfulness of experiences in the present moment, 2) didactic teaching on mindfulness, stress, stress management and mindful communication, using a course manual and CDs for home practice, and 3) a group process to facilitate reflections on practising mindfulness both at home and during classes. The instructors focused on creating an open, curious, non-judgemental and accepting attitude towards all participant experiences. The course manual used in this study is available on request.

### Instructor qualifications and compliance with the MBSR manual

The instructors (three men and three women) were trained in conducting MBSR courses and had practiced mindfulness for many years. Both project managers received their instructor training provided by the Center for Mindfulness in Massachussets, USA, and were in agreement regarding the content and format of the MBSR course manual. When running the first course they also consulted each other after every class to ensure programme fidelity.

### Measures

In addition to the information gathered about participant age, gender, marital status (coded as ‘single’ or ‘living with partner’) and how many children they had (coded as ‘none’ or ‘having children’), outcome measures were chosen that would capture the possible intervention effects on different aspects of psychological health, including mental distress, study stress, student burnout, subjective well-being, and mindfulness. We also measured student compliance as indicated by course attendance and self-reported home practice.

*Mental distress* was measured using the 12-item General Health Questionnaire (GHQ12) [29]. This consisted of questions related to participant mental distress experience in the last two weeks and used four evaluation response categories: ‘more than usual’ (0), ‘same as usual’ (1), ‘less than usual’ (2), and ‘much less than usual’ (3). The total possible score ranged from 0 (no distress) to 36. The Cronbach’s alpha value for our sample was .90. The GHQ12 response categories were further dichotomised, with ‘0’-‘1’ evaluations scored as ‘0’ while ‘2’-‘3’ evaluations were scored as ‘1’. A cut-off score of ‘≥4’ indicated a clinically significant level of mental distress [23].

*Student burnout* was measured using a version of the 15-item Maslach Burnout Inventory (MBI) tailored to measure three dimensions of student burnout, namely: emotional exhaustion (5 items), cynicism (4 items), and study efficacy (6 items) [30]. The items had seven response categories ranging from ‘never’ (0) to ‘always’ (6). A summed score was calculated based on a reversal of the efficacy items and evaluated on a scale ranging from 0 (indicating ‘no burnout’) to 90. The MBI inventory is cross-culturally valid, has good psychometric properties [30], and has been tested on pre-clinical and clinical medical students [31]. In our sample, the Cronbach’s alpha value for the sum scale was .90.

*Study stress* was measured using the 13-item Perceived Medical School Stress (PMSS) scale [32], with one item adapted for cultural reasons [33]. The PMSS assessment has been shown to have adequate predictive validity for mental health problems in medical professionals four years after graduation [34]. In our study, the PMSS was adapted by removing the word ‘medical’ in all instances of the term ‘medical study’. The 13 items had five response categories which ranged from ‘strongly disagree’ (0) to ‘strongly agree’ (4), and the total sum score ranged from 0 (indicating ‘no stress’) to 52. The Cronbach’s alpha value for our sample was .79.

*Subjective well-being (SWB)* was measured using a 4-item version of the SWB scale [35]. Previous use of this scale has indicated that is has good psychometric properties and correlates strongly and positively with the Satisfaction With Life Scale [36]. In accordance with generally accepted dimensions of well-being scales [36], the SWB construct consists of a cognitive element (life satisfaction), a positive affect element (happy and strong) and a negative affect element (unhappy and tired). The number of the response categories varied and therefore all items were transformed to a scale ranging from 0–10, using the algorithm: X = (Y-1) × 10/(Z-1), where X is the new score, Y the original score, and Z the number of response categories. Higher scores reflect increased subjective well-being. The Cronbach’s alpha value for our sample was .81.

*Mindfulness* was measured using the Five Facet Mindfulness Questionnaire (FFMQ; 39 items). This questionnaire has been shown to have good psychometric properties [37] and was used in our study to measure five facets of mindfulness*.* The Norwegian version of the questionnaire was translated using a standard forward-backward process at the University of Bergen and has also been used in a recent Norwegian MBSR study [38]. The first four facets consisted of eight items each, while the fifth had seven items. Each item had five response categories which ranged from ‘never or very seldom true’ (1) to ‘very often or always true’ (5). In our sample, the five facets (and corresponding Cronbach’s alpha values) were: the ability to a) observe (.78), b) describe (.89), c) act with awareness (.88) together with the ability to be fully present with an attitude of d) non-judgement (.92), and e) non-reactivity (.73) towards what occurs. Suboptimal properties of the non-reactivity facet in a student sample have also been found in previous research [37]. In student populations the FFMQ is positively correlated with meditation experience, openness to experience, emotional intelligence and self-compassion. It is also strongly negatively correlated with psychological symptoms, neuroticism, thought suppression and difficulties in emotional regulation [37]. Higher scores indicate increased mindfulness.

*Student compliance* measured attendance and self-reported home-based mindfulness practice. *Attendance* was measured by the number of classes attended (0-7). *Mindfulness practice* was assessed using four questions: a) ‘How often have you practised mindfulness exercises (body-scan, relaxation, yoga, gi gong, tai chi or meditation) in the last four weeks?’ (the six response categories ranged from ‘never’ (0) to ‘daily’ (5)); b) ‘When you practise, how long do you normally practise?’ (six response categories which ranged from 0 minutes (0) to >45 minutes (5)); c) ‘How often have you practised mindful breathing in the last four weeks?’ (six response categories which ranged from ‘never’ (0) to ‘daily’ (5)), and d) ‘How often have you practised being mindful in everyday situations in the last four weeks?’ (six response categories which ranged from ‘never’ (0) to ‘daily’ (5)). Mindfulness practice was measured as a summed score (ranging from 0 to 20).

### Statistical analyses

The success of the randomisation procedure was evaluated by analysing T1 mean score differences between the intervention and control groups using independent sample t-tests and chi-square test for categorical variables. Completer and dropout comparisons were also examined using the same tests. The online questionnaire was constructed in a way that ensured that all items on each page had to be completed before respondents were able to progress to the next page. Instances of missing data were caused by discontinuation of the questionnaire (one student) or a loss of respondents to follow-up (eleven students). Data were missing from the responses of five students in the intervention group and seven in the control group respectively. The last-observation-carried-forward method of imputation was chosen as this is a conservative method used in instances in which there is an equal drop-out rate in the intervention and the control group [39]. Intention-to-treat analyses and per protocol analyses yielded very similar results and we have therefore presented only the former.

Multivariate analyses of covariance (MANCOVA) were applied to the multiple dependent variables measured at T2 (i.e. mental distress, student burnout, study stress, and subjective well-being). Analyses of covariance (ANCOVAs) were then applied. T1 measures were included as covariates because the correlation coefficients between the measurements at T1 and T2 were substantial. The use of covariate control increased the statistical strength of the results by reducing unexplained or error variance. This same approach was used to examine the effect of the intervention on the five facets measuring mindfulness. As gender had not been accounted for by stratified randomisation, this was included as a factor in the MANCOVA analysis in order to estimate its effect on the intervention. Alpha-levels were adjusted for multiple testing by applying a Bonferroni correction.

A linear regression analysis was used to test the relationship between MBSR attendance and mindfulness practice and the outcome variables. Multilevel mixed linear regression analyses were conducted to investigate whether MBSR effects depended on the student class (five medicine and five psychology classes as random factors) or the university locations (Oslo and Tromsø as fixed factors). The study instructors varied by university location and these factors therefore coincided. Mediation analyses will be conducted following collection of two-year follow-up data.

Hedges’ *g* was used to calculate the size of the treatment effect by estimating the standardised mean difference in test scores between the intervention and control group (Tables 1 and 2). Hedges’ *g* is similar to Cohen’s *d* (with a pooled SD) but has slightly improved precision as the result of the inclusion of a correction factor for small sample sizes. The two effect-sizes are related accorded to the equation $g=\sqrt{\frac{n_{1}+n_{2}-2}{n_{1}+n_{2}}}d$, and the values used for interpreting effect size are 0.2 (small), 0.5 (moderate) and 0.8 (large) [40]. We calculated the Number Needed to Treat (NNT) which was used as a measure to assess the clinical importance of the effect found on mental distress. NNT is defined as the expected number of people that need to receive an intervention rather than the control condition for one additional person to have a specified effect within a given time frame [41].

**Table 1:** Outcome measures at T1 and T2 for the intervention and control group

	**Intervention *n *=144**		**Control *n *=144**			
	**Women *n*** **= 118**		**Women *n*** **= 101**			
	**Men *n*** **= 26**		**Men *n*** **= 43**			
	**T1**	**T2**	**T1**	**T2**	**Hedges’ g**	***F***_**1,287**_
					**(95% CI)**	**Women *F***_**1,218**_
						**Men *F***_**1,67**_
						**( *p- *value)**
**GHQ12**	**12.4 (6.0)**	**9.2 (4.0)**	**13.0 (6.2)**	**13.2 (6.1)**	**0.65 (.41, .88)**	**44.55 (<.001)**
Women	12.8 (5.9)	9.2 (4.1)	13.9 (6.3)	14.1 (6.1)	0.72 (.45, .99)	47.21 (<.001)
Men	10.8 (6.1)	9.3 (3.4)	11.0 (5.6)	11.1 (5.6)	0.33 (−.16, .82)	2.28 (.136)
**Burnout**	**32.3 (12.4)**	**32.9 (12.1)**	**32.0 (11.8)**	**34.4 (11.2)**	**0.15 (−.08, .38)**	**1.63 (.204)**
Women	32.2 (12.9)	32.7 (11.9)	32.5 (12.1)	35.3 (11.9)	0.19 (−.08, .46)	3.69 (.056)
Men	32.5 (14.0)	33.9 (13.1)	30.7 (11.0)	32.4 (9.3)	0.02 (−.47, .51)	0.08 (.779)
**PMSS**	**18.9 (6.9)**	**18.4 (6.8)**	**19.5 (7.0)**	**20.3 (7.4)**	**0.17 (−.07, .40)**	**5.38 (.021)**^**a**^
Women	19.1 (6.8)	18.3 (6.5)	20.6 (7.3)	21.6 (7.9)	0.25 (.02, .52)	9.58 (.002)
Men	17.6 (7.4)	18.9 (7.9)	16.9 (5.6)	17.1 (5.2)	0.17 (−.32, .66)	1.09 (.300)
**SWB**	**6.3 (1.8)**	**6.8 (1.4)**	**6.4 (1.8)**	**6.1 (1.8)**	**0.40 (.27, .63)**	**16.16 (<.001)**
Women	6.3 (1.7)	6.8 (1.4)	6.2 (1.8)	5.8 (1.9)	0.61 (.34, .88)	32.15 (<.001)
Men	6.4 (2.1)	6.3 (1.5)	6.8 (1.7)	6.9 (1.5)	0.19 (−.30, .68)	1.88 (.175)

*Note.* Means (SD), *g* between group Hedges effect sizes and *p*-values from univariate tests across gender. *CI* Confidence Interval based on pooled post-intervention SD. Bold characters reflect data for the whole sample.

^a^Did not reach significance using a Bonferroni-corrected alpha-level of 0.0125.

**Table 2:** Outcome on 5 mindfulness measures at T1 and T2 for the intervention and control group

	**Intervention *n *=144**		**Control *n *=144**			
	**Women *n*** **= 118**		**Women *n*** **= 101**			
	**Men *n*** **= 26**		**Men *n*** **= 43**			
	**T1**	**T2**	**T1**	**T2**	**Hedges’g**	***F***_**1,287**_
					**(95% CI)**	**Women *F***_**1,218**_
						**Men *F***_**1,67**_
						**( *p- *value)**
**Non Reacting**	**20.5 (3.8)**	**21.9 (3.6)**	**20.4 (3.9)**	**20.7 (4.0)**	**0.33 (.10, .56)**	**10.70 (<.001)**
Women	20.4 (3.7)	21.9 (3.7)	20.2 (4.0)	20.7 (4.2)	0.27 (.00, .54)	6.78 (.010)
Men	21.2 (4.1)	22.2 (2.8)	20.8 (3.7)	20.8 (3.4)	0.32 (−.17, .81)	3.22 (.077)
**Non Judging**	**25.4 (5.6)**	**26.4 (5.2)**	**25.9 (5.4)**	**26.4 (5.2)**	**0.17 (−.06, .40)**	**2.98 (.085)**
Women	25.3 (5.9)	26.9 (5.4)	25.3 (5.6)	25.5 (5.5)	0.27 (.00, .54)	7.31 (.007)
Men	25.9 (5.2)	26.5 (4.3)	27.2 (4.7)	28.7 (3.9)	0.21 (−.28, .70)	3.70 (.059)
**Act Aware**	**23.8 (5.2)**	**24.4 (4.6)**	**24.8 (5.9)**	**24.6 (5.5)**	**0.15 (−.08, .38)**	**1.02 (.314)**
Women	24.0 (5.0)	24.5 (4.62)	24.4 (5.5)	23.8 (5.6)	0.18 (−.09, .45)	3.492 (.063)
Men	23.4 (6.0)	24.0 (4.8)	25.9 (6.0)	26.4 (4.8)	0.02 (−.47, .51)	1.293 (.290)
**Describe**	**28.6 (5.6)**	**29.6 (5.3)**	**29.3 (5.1)**	**29.9 (5.2)**	**0.06 (−.17, .29)**	**0.13 (.719)**
Women	28.5 (5.7)	29.6 (5.2)	29.2 (5.1)	30.2 (5.7)	0.03 (−.24, .30)	.000 (.987)
Men	29.2 (5.4)	29.5 (5.9)	29.4 (5.3)	29.4 (3.8)	0.07 (−.42, .56)	.052 (.820)
**Observe**	**26.7 (5.0)**	**27.4 (5.1)**	**26.7 (5.3)**	**26.4 (5.7)**	**0.17 (−.06, .40)**	**4.54 (.034)**^**a**^
Women	27.0 (5.2)	27.6 (5.2)	27.1 (5.1)	26.8 (5.6)	0.14 (−.13, .41)	2.334 (.128)
Men	25.5 (3.9)	26.5 (4.2)	25.6 (5.7)	25.3 (5.9)	0.25 (−.24, .74)	1.946 (.168)

*Note.* Means (SD), *g* between group Hedges effect sizes and *p*-values from univariate tests across gender. *CI* Confidence Interval based on pooled post-intervention SD. Bold characters reflect data for the whole sample.

^a^Did not reach significance using a Bonferroni-corrected alpha-level of 0.01.

## Results

### Study flow and attrition

**Figure 1:**
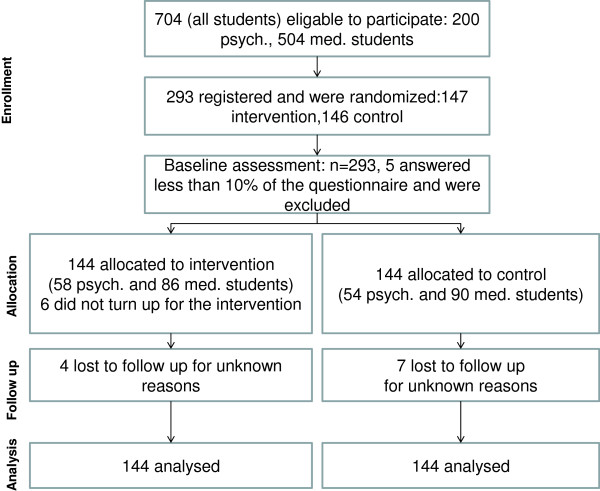
Flowchart describing recruitment and dropout.

Figure 1 illustrates the study participant flow. An analysis of participant drop-out indicated no significant differences in the demographic data or the outcome measurements at T1 between those subjects participating and those dropping out at T2. There were no reported harms or unintended effects of the intervention. Some students reported that they experienced adverse emotional, mental or bodily states during mindfulness practice. However, this was not considered to be unintended effects of the intervention, but rather expected results of becoming more mindful of inner experiences.

### Descriptive analyses and randomisation check

There were no significant differences between the intervention and control group on the outcome measures or the demographic variables at T1, except for gender (Table 3). Demographic variables and outcome measures at T1 did not differ by study subject (medicine or psychology) or study location (Oslo or Tromsø). The level of mental distress in our study was high, and 25% of the men and 36% of the women scored above the GHQ12 cut-off score (i.e. ≥4). The gender difference in mental distress was significant (*χ*^*2*^ *=* 5.58, *p* = .02). Compared with men, women also scored higher on study stress (*F*_1,287_ *=* 8.08*, p <* .01) and on the mindfulness facet ‘observe’ (*F*_1,287_ = 4,62, *p* < .05). Table 1 and Table 2 outline all descriptive data for the measurements at T1 and T2 for the intervention and control groups respectively.

**Table 3:** Socio-demographic characteristics of the intervention and control group at T1

**Characteristic**	**Overall**	**Intervention**	**Control**	***p-v*alue**
	***N*** **= 288**	***n*** **= 144**	***n*** **= 144**	
Mean age (SD)	23.8 (5.2)	23.6 (4.7)	24 (5.7)	.58
Women, N (%)	219 (76)	118 (82)	101 (70)	.03
University, N (%)				.63
Oslo	179 (62)	87 (60)	92 (64)	
Tromsø	109 (38)	57 (40)	52 (36)	
Study, N (%)				.72
Medicine	176 (61)	86 (60)	90 (62)	
Psychology	112 (39)	58 (40)	54 (38)	
Civil status, N (%)				.16
Married/cohabiting	86 (30)	37(26)	49 (34)	
Single	202 (70)	107 (74)	95(66)	
No of children, N (%)				.34
0 children	269 (93)	137 (95)	132(92)	
1-5 children	19 (7)	7 (5)	12 (8)	

### Effects of the intervention on the main outcome measures

The MANCOVA analysis revealed a significant overall effect on the main outcome measures of the intervention compared with the control group (*F*_1, 287_ = 12.06, *p* < .001). Follow-up univariate ANCOVA analysis showed a significant effect of the intervention on mental distress and well-being. The intervention did not significantly reduce student stress or student burnout (Table 1). The number of students scoring below a cut-off score of ≥4 on GHQ12 at T2 was 128 in the intervention and 84 in the control group. We calculated an absolute risk difference of 0.31 and a NNT = 1/0.31 = 4.

**Figure 2:**
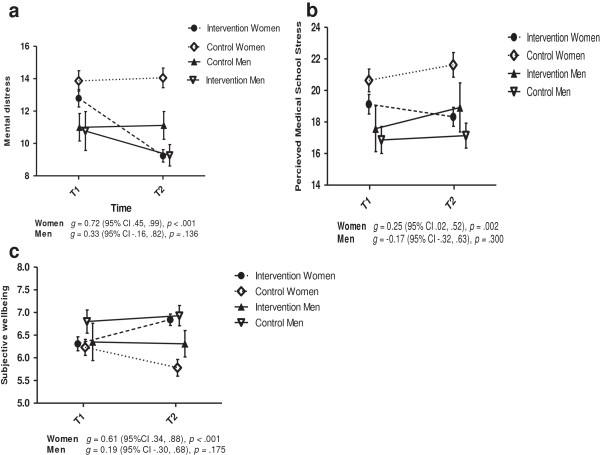
**Gender effects of MBSR intervention (means, SD) on mental distress (Figure **2**a), perceived medical school stress (Figure **2**b) and subjective wellbeing (Figure **2**c) including means and SD.**

When gender was included as a factor in the MANCOVA analyses of the main outcomes, the effect of the intervention remained significant (*F*_1, 287_ = 6.64, *p* < .001) and, in addition, the interaction effect of group × gender was significant (*F*_1,287_ *=* 5.34, *p* < .001). Follow-up ANCOVA analyses indicated that the intervention had a significant effect for women on mental distress, subjective well-being and student stress, but not for men (Table 1). The direction of the effects is illustrated in Figure 2. Women also showed a reduction in burnout in the expected direction (*F*_1,287_ *=* 3.69, *p* = .056).

### Effect of the intervention on the mindfulness facets

A MANCOVA analysis with the five mindfulness facets at T2 as dependent variables and their corresponding T1 measurements as covariates showed an overall significant effect in favour of the intervention group (*F*_1,287_ = 3.10, *p <* .01). Using a Bonferroni-corrected alpha-level of .01, follow-up analyses showed that the effect was only significant on the non-reactive mindfulness facet scores (Table 2). Adding gender as a between-group factor did not reveal any interaction between group and gender, but separate analyses for gender indicated that the effect for female students was also significant on the mindfulness facet ‘non-judging’ (Table 2).

### Effects of study, university location, course instructor, mindfulness practice and attendance on the outcome measures

Multilevel mixed linear regression analyses indicated that the intervention effects on mental distress and well-being did not vary by university location, course instructors, student class or the type of study.

Men and women attended the intervention group and practised mindfulness to the same degree (ANOVA, *F*_1,143_ = 1.26*, p* = .26 for attendance and*, F*_1,143_ = 0.74, *p* = .39 for practice). The average attendance rate was 5.3 (SD 1.9) out of seven sessions. The students in the intervention group reported undertaking formal practice 1.5 times a week on average, with an average duration of 13 minutes per session. The degree of attendance and sum of the duration of the home practice of mindfulness were significant moderators of the treatment effect in terms of mental distress at T2 when controlling for mental distress at T1 and gender. More exercise (*β* = .24, *p* < .05) and more attendance (*β* = .25, *p* < .01) were associated with increased intervention effect. The degree of exercise also predicted levels of the non-reactive mindfulness facet (*β* = .33, *p* < .001). The other outcome measures were not significantly moderated by attendance and mindfulness practice.

## Discussion and conclusions

As hypothesised, the seven-week course in mindfulness training reduced mental distress and improved student well-being independent of the student classes (medicine or psychology), university locations (Oslo and Tromsø), and course instructors. The intervention had no statistically significant effect on student burnout. Only female students showed a significant intervention effect on mental distress, study stress and well-being. A higher level of class attendance and mindfulness practice at home increased the effect of the intervention, particularly for mental distress. The intervention increased the ability of female students to be mindful with acceptance and not to react automatically to internal and external stimuli.

Our findings concur with other studies of students which have reported similar increases in positive states of mind as a result of MBSR interventions [16,19]. Reductions in mental distress and improved well-being in medical students have been observed previously in randomised mindfulness intervention studies [15,17]. However, the current study is the first randomised controlled trial to show that a mindfulness intervention can reduce mental distress and study stress and increase subjective well-being in medical *and* psychology students. It is also the first study to demonstrate that an MBSR intervention for students may work within a non-USA cultural setting. Further, our study is the first randomised controlled study to report on differential gender effects of participating in an MBSR intervention.

The effect of the course on mental distress was moderate and is in keeping with findings from other controlled MBSR student intervention studies. Jain et al. (2007), for example, noted large effect sizes on mental distress, rumination and positive states of mind following a four-week MBSR course for medical and nursing students [17], while Shapiro et al. (1998) noted moderate effect sizes on mental distress, anxiety and depression following a seven-week MBSR course for medical students [15]. Our study reported a NNT value of 4 which is a measure of the practical relevance of our intervention on mental distress. This NNT indicates that in order to move one student from above to below the cut-off score for mental distress, four students would need to receive the intervention.

To our knowledge, only two controlled studies have previously investigated the impact of gender on the effect of the MBSR programme [24,42]. Both included adult populations and reported equal gender effects. In a review of gender differences in the effect of MBSR treatments for substance abuse disorders, two papers based on one controlled trial found no gender-specific effects, and two quasi-experimental studies indicated a larger benefit among women [43]. Our study showed a gender difference in the effect of the MBSR intervention in favour of women. Although men did experience a small effect on mental distress in our study, this effect was not statistically significant, possibly due to the fact that there were significantly fewer men in the intervention group than the control group.

At T1, women reported higher study stress and mental distress, a finding which has been previously reported [22,23]. Such gender differences in reporting distress may be related to biological processes related to how stress and emotions are *experienced*[44] as well as gender-specific socialisation processes associated with how stress and emotions are *expressed*[45,46]. The seven weeks of mindfulness practice may have helped male students to become more *aware* of their distress, but may have assisted female students with *handling* their distress better. These findings suggest that men may need more extensive – or different forms – of mindfulness training in order to obtain satisfactory benefits. However, our finding could also be specific to students and due perhaps to differences in maturity specific to this age range. Future qualitative interviews with the male students who participated in the study may shed further light on this issue.

Interestingly, women at T1 scored higher on the ‘observe’ facet of mindfulness. For students who do not practise mindfulness, the ability to observe is inversely correlated with mental health measures [37]. By learning mindfulness, student mental health can be enhanced through an improved ability to observe *with* an acceptance that is non-judging and non-reacting [47]. Our findings are similar to these earlier results given that the female students reported both enhanced mental health *and* scored significantly higher on the ‘non-reacting’ and ‘non-judging’ facets of mindfulness after the intervention. These findings are further supported by research showing the importance of these two facets of mindfulness on the effect of the intervention [48].

Course attendance and the home practice of mindfulness moderated the intervention effects on mental distress but did not affect subjective well-being. Several studies however have reported inconsistent results regarding the relationship between student compliance (attendance and practice) and outcome [14,49] ranging from no correlation [17] to a positive correlation [15].

Recently, several mediation analysis studies have supported a causal relationship between increased mindfulness and positive health outcomes [24,38,50] and this finding will be tested in a two-year follow-up of our study. However, we have found only small effect sizes for mindfulness, and the level of mindfulness measured at T2 is considerably lower than those reported in studies of experienced meditators [48]. This may be due to the low levels of formal home practice reported by the students. Whether additional practice could result in increased levels of mindfulness will be evaluated in our follow-up studies. We still do not fully know how mindfulness practice works or the specific individual characteristics that help to promote the effects of MBSR. Different people may, for instance, need different amounts and types of practice. That only practice rather than attendance per se was a predictor of variation in the ‘non-reacting’ facet of mindfulness may indicate that the degree to which one practises mindfulness is a plausible key to understanding the effects of the intervention. The reason why attendance and practice did not predict changes in well-being is difficult to explain and future studies are needed to explore this issue in greater depth.

The research strength of this study was enhanced in a number of ways, including the use of a computer-randomised controlled design, concealment of allocation, an electronic assessment of the outcomes which remained free of the influence of the study evaluators, and the low level of sample attrition. Also, the fact that the effects were found irrespective of the student classes, study sites, and course instructors makes it possible to assume that the effects were due to the mindfulness intervention itself. A broader intervention strategy may have enabled more students to participate.

The limitations of the study include a possible selection bias during recruitment which may have affected the results. As only 40% of the eligible students volunteered to participate, those students who were recruited might have been more motivated to take part and possibly more primed to focus on psychological and personal issues. In addition, because the active ingredients of the intervention are “transportable” and participants from the intervention group and the control group interacted during and after the intervention period, contamination may have occurred, which may have influenced the magnitude of the effect sizes. Moreover, because the study randomisation was not stratified for gender, only 26 men received the intervention. Necessarily, this resulted in insufficient statistical strength and inconclusive interpretations regarding the impacts of the intervention on male students. The study did also not include a comparable control intervention in which the same amount of attention from instructors and regular participation was provided within a supportive group of fellow students. Thus we are unable to specify which particular elements of the intervention may have been more strongly associated with the resultant outcomes. Participants were also not asked to keep daily logs of their mindfulness exercises, and it’s possible that such records may have helped to shed light upon the impact of the exercise on outcomes. The suboptimal property of the non-reactivity facet of mindfulness has also limited our conclusions related to the mindfulness effect of the intervention. Finally, adherence to the MBSR manual was not systematically evaluated in terms of, for example, the use of video or audio recordings during the intervention sessions.

In conclusion, the present study shows that teaching medical and psychology students to relate mindfully to current internal and external stimuli can decrease mental distress and increase well-being. There is a need for more research on mindfulness-based interventions that includes gender as a variable. The degree to which this MBSR intervention will influence mental distress and subjective well-being in the students’ later years of studies and in their professional career is a research question that will be addressed in our follow-up studies.

## Competing interest

The authors declare that they have no competing interests.

## Authors’ contributions

MdV, AB and RT defined the research theme and designed the study. IS, JR and TS helped in the design of the study. MdV and IS were responsible for recruitment, intervention delivery and the acquisition of data, and they analysed and interpreted the data and drafted the manuscript. AB, RT, JR and TS helped to interpret the data and revise the manuscript critically for important content. OF helped with the statistical analyses and the interpretation of the data and revised the manuscript critically. All authors read and approved the final manuscript.

## Pre-publication history

The pre-publication history for this paper can be accessed here:

http://www.biomedcentral.com/1472-6920/13/107/prepub
